# Exploration of plant genomes in the FLAGdb^++ ^environment

**DOI:** 10.1186/1746-4811-7-8

**Published:** 2011-03-29

**Authors:** Sandra Dèrozier, Franck Samson, Jean-Philippe Tamby, Cécile Guichard, Véronique Brunaud, Philippe Grevet, Séverine Gagnot, Philippe Label, Jean-Charles Leplé, Alain Lecharny, Sébastien Aubourg

**Affiliations:** 1 Unité de Recherche en Génomique Végétale (URGV), UMR INRA 1165 - Université d'Evry Val d'Essonne - ERL CNRS 8196, 2 Rue Gaston Crémieux, CP 5708, F-91057 Evry Cedex, France; 2 Unité Mathématique Informatique et Génome (MIG), UR INRA 1077, Domaine de Vilvert, F-78352 Jouy-en-Josas Cedex, France; 3 Laboratoire de Chimie Bactérienne (LCB), UPR CNRS 9043 - IFR 88, 31 Chemin Joseph Aiguier, F-13009 Marseille, France; 4 Unité Amélioration, Génétique et Physiologie Forestières (UAGPF), UR INRA 588, 2163 avenue de la Pomme de Pin, CS 4001 Ardon, F-45075 Orléans, France

## Abstract

**Background:**

In the contexts of genomics, post-genomics and systems biology approaches, data integration presents a major concern. Databases provide crucial solutions: they store, organize and allow information to be queried, they enhance the visibility of newly produced data by comparing them with previously published results, and facilitate the exploration and development of both existing hypotheses and new ideas.

**Results:**

The FLAGdb^++ ^information system was developed with the aim of using whole plant genomes as physical references in order to gather and merge available genomic data from *in silico *or experimental approaches. Available through a JAVA application, original interfaces and tools assist the functional study of plant genes by considering them in their specific context: chromosome, gene family, orthology group, co-expression cluster and functional network. FLAGdb^++ ^is mainly dedicated to the exploration of large gene groups in order to decipher functional connections, to highlight shared or specific structural or functional features, and to facilitate translational tasks between plant species (*Arabidopsis thaliana*, *Oryza sativa*, *Populus trichocarpa *and *Vitis vinifera*).

**Conclusion:**

Combining original data with the output of experts and graphical displays that differ from classical plant genome browsers, FLAGdb^++ ^presents a powerful complementary tool for exploring plant genomes and exploiting structural and functional resources, without the need for computer programming knowledge. First launched in 2002, a 15^th ^version of FLAGdb^++ ^is now available and comprises four model plant genomes and over eight million genomic features.

## Background

Holistic approaches require the organization of data and metadata in order to allow the hypothesis-driven querying of heterogeneous objects. In many systems biology considerations, data management and integrative approaches are identified as key to the thorough exploitation of omics data and their translation into knowledge [1]. Many biologists that would like to take advantage of the rapid increase in the number and size of sequenced genomes do not have the skills required to derive function from sequence or vice versa. They encounter a major problem, *i.e. *connecting heterogeneous pieces of information quickly and accurately in the absence of a methodological approach to organizing them efficiently. Indeed, huge quantities of data are stored and managed by different databases, but linking this information is highly complex [2]. This is particularly true when users with no computer programming skills wish to retrieve a large set of information from a list of tens or hundreds of genes, a frequent case nowadays since the advent of different omics approaches. For instance, a transcriptomics experiment yields large lists of differentially expressed genes dependent on two alternative conditions and researchers need to know as much information as possible about them in order to progress to the next step in a hypothesis-driven process. The same applies to proteomics or interactomics approaches. Thus, it helps greatly to use a tool that quickens this task whilst providing highly accurate results. FLAGdb^++ ^is designed to be such a tool, efficiently navigating in and between plant model genomes in order to analyze large sets of genes. The main design criteria included (i) using a common information system for all genomes within a unified interface, (ii) providing reliable data by combining and re-analyzing raw data derived from different sources, *i.e. *ridding users of format heterogeneity problems, (iii) considering data in various contexts such as chromosomal location, or gene family or orthology group membership, (iv) providing access to original data through collaboration with data producers, and (v) facilitating the formulation and testing of hypotheses based on links between gene structure and function. In order to satisfy these criteria, the choice was made to develop a data warehouse connected to original interfaces and capable of helping build hypotheses based on a number of interactive graphical displays. Deciphering the functional relevance of a gene cluster and inferring hypothesis from common characteristics are both complex processes involving multiple information sources, steps and queries which may not necessarily be fully predictable at the start. In FLAGdb^++^, the graphical displays are centered on highly connected map-like representations, intended to act together as mnemonics to guide hypothesis establishment progression. When initially launched in 2002 FLAGdb^++ ^focused solely on the *Arabidopsis thaliana *genome [3], but has now expanded to incorporate other plant genomes and is involved in an increasing number of genomic projects. Due to close collaboration with biologists, data producers and experts in genomic resources, the development and improvements made to FLAGdb^++ ^allow the clear presentation of original data, thanks to an intuitive graphical tool box. Beyond the adding of novel data types and cross-references, the new functionalities allow the users to compare gene structures and promoters, and to navigate into gene classification, segmental duplications, feature density curves, phylogenetic profiles and orthology groups. Finally, FLAGdb^++ ^efficiently completes other plant genome databases and browsers [4-7].

## Construction and content

### Architecture

FLAGdb^++ ^is based on a client-server model. The n-tier architecture is composed of a relational database (under RDBMS PostgreSQL) and a client application, implemented in JAVA (JDK 1.6), and contains the application server and user interfaces. Communication with the database relies on the JDBC driver. The client application has to be locally installed by the users in order to query the FLAGdb^++ ^database through the graphical interfaces. The JAVA WEB START technology is used to facilitate and automate the installation and updates of the application. The JAVA solution has been selected for its compatibility with all operating systems (JAVA Runtime Environment is now available by default on almost all computers) and to enhance the possibilities of development around the user-side application. Concerning the database, the schema has been designed to scale well with very large quantities of diverse data, allowing the connection of features and information not only around genomic loci, but also around biological functions or gene families. Thus, this architecture proves a good compromise between performance, scalability and development issues.

### Data

FLAGdb^++ ^has been developed in a generic way in order to be applied to different genomes. Therefore, it is able to store, organize, explore and analyze numerous types of genomic resources (called features). Data integration is based on mapping to genomic sequences using the genomic coordinates as an index system. The database schema and interfaces consider different types of data along with their origin, quality and biological relevance, and the diversity of possible queries in order to access and analyze them.

In addition to the *Arabidopsis thaliana *genome (Columbia 0, [8]) FLAGdb^++ ^now contains the genomes of *Oryza sativa *(spp japonica cv. Nipponbare [9]), *Populus trichocarpa *(Nisqually-1 clone [10]) and *Vitis vinifera *(PN40024, 12x assembly [11]). These four complete plant genomes, representing four distinct angiosperm taxa in the plant kingdom, are stored in the same database instance and can be queried using the same tools within the FLAGdb^++ ^application.

Beyond the basic genome-wide annotation of CDS, FLAGdb^++ ^aims to merge different genomic resources in order to improve the structural and functional annotation of genomes. These resources derive from several origins: general or specific databases, internal and collaborative projects, experimental high-throughput approaches, manual biocuration or *in silico *prediction works (Table 1). The diversity and quality of features and annotations vary between species due to unequal community sizes and the time elapsed since the end of the sequencing project. The integration task involves several steps of selection, expertise and possible enrichment through data post-processing, filtering (with quality cut-off) and additional predictions. For example, with the aim of having an homogeneous overview, the functional annotation of all protein-coding genes (from the four genomes) has been completed by (i) the prediction of targeting signals by a unique pipeline combining Predotar [12], WoLF PSORT [13] and CBS tools [14] and (ii) the definition of phylogenetic profiles based on the presence or absence of homologs in 11 different phyla. For Arabidopsis, secondary and 3 D structures have been predicted from primary protein sequences and local similarities in PDB proteins [15,16] with such results constituting an original resource for functional insights and being complementary to another similar initiative based on different method [17]. Also concerned with data improvement, which is of central interest to FLAGdb^++^, all the transcript sequences available in GenBank/dbEST are consistently mapped on and spliced-aligned against integrated genomes. Results are then exploited to redefine the 5' and 3' UTR extremities of each transcriptional unit. The deduced new transcription start sites allow for better definition of promoter regions and further help to characterize motifs of biological relevance [18]. Indeed, FLAGdb^++ ^is more than a collection of data since the genomic resources are carefully selected, verified, improved, completed and finally integrated in order to increase both their complementarity and biological content. FLAGdb^++ ^constitutes a significant step in transforming data into knowledge.

**Table 1:** List of genomic data available in FLAGdb^++^

Data type	Feature number	Sources
***Arabidopsis thaliana***		
AGI coding genes	28 094	TAIR [21,39]
EuGène coding genes	27 981 *	[19,20]
RNA genes	1 288	TAIR [39] and miRbase [40]
Transposable elements	3 900	TAIR [39]
Curated repeat elements	31 876 *	[36]
Transcript sequences (EST, cDNA)	1 281 393	GenBank, aligned with SIM4 [41]
Predicted smallRNA genes	609 *	O. Voinnet *et al. *(unpublished data)
2 D structures	24 194 *	Predicted by SOPMA, PHD, DSC [15]
3 D structures	8 492 *	Predicted by Geno3 D [16]
Curated annotations	2 728 *	[33,34,42]
Paralogs in duplicated segments	14 228	TIGR-JCVI [43]
FST	407 192	INRA, GABI, SAIL and SALK [44]
CATMA probes (GST and GFT)	35 283 *	CATMA and CATdb [24,25,45]
Affymetrix micro-array probes	266 372	GeneChip^® ^Arabidopsis ATH1
Chr. 4 tiling-array probes	21 752 *	[26]
Whole genome tiling-array probes	1 434 492 *	TAG project (unpublished data)
Promoter-array probes	11 904 *	SAP project [27]
MPSS from mRNA and smallRNA	136 407	Arabidopsis MPSS plus [46,47]
Gene families	3 500	PFAM profiles [32]
Protein motifs	38 631	PFAM profiles and HMMER [48]

***Oryza sativa***		
Coding genes	41 439	TIGR-JCVI and RAP-DB [49,50]
RNA genes	718	TIGR-JCVI [49]
Repeat elements	16 185	TIGR-JCVI [49]
Transcript sequences (EST, cDNA)	1 120 229	GenBank, aligned with SIM4 [41]
Curated annotations	477 *	[35]
FST	79 612	OryGenesDB [51]
Gene families	2 988	PFAM profiles [32]
Protein motifs	60 789	PFAM profiles and HMMER [48]

***Populus trichocarpa***		
Coding genes	45 555	JGI [10]
Repeat elements	29 366	JGI [10]
Transcript sequences (EST, cDNA)	322 996	GenBank, aligned with SIM4 [41]
Curated annotations	3 176 *	J.-C. Leplé *et al. *(unpublished data)
Gene families	3 371	PFAM profiles [32]
Protein motifs	49 723	PFAM profiles and HMMER [48]

***Vitis vinifera***		
IGGP coding genes	26 347	Genoscope [11] using GAZE [52]
EuGène coding genes	44 414 *	[19]
Repeat elements	336 729	Genoscope [11]
Transcript sequences (EST, cDNA)	419 542	GenBank, aligned with SIM4 [41]
Curated annotations	220 *	TPS [22] and other unpublished families
Gene families	2 970	PFAM profiles [32]
Protein motifs	32 375	PFAM profiles and HMMER [48]

*: original data, only in FLAGdb^++^

For both Arabidopsis and the grapevine, we have completed the structural annotation of the genomes using an additional genome-wide prediction of CDS via the predictor-combiner software EuGène [19]. The relevance of hundreds of genes previously only predicted by EuGène has now been ascertained using transcriptomic and sequencing data [20] and they are now recognised by TAIR [21]. For *Vitis vinifera *also, previous manual annotation of gene families validates the complementary contribution of EuGène in the structural annotation of the genome [22]. This illustrates one of the roles that a specific intermediate database such as FLAGdb^++ ^may play in providing access to original new resources to the community for their deep analyses and expertises before release, after validation, into renowned large repositories.

The EuGène results have also been used, in a complementary manner to AGI annotation work, to design the probes for different versions of the CATMA micro-array [23,24]. Beside Affymetrix ATH1 GeneChips, CATMA micro-arrays provide a significant amount of transcriptome data covering a large spectrum of physiological conditions and mutants [25]. FLAGdb^++ ^is used as a repository for different kinds of CATMA probes, *i.e. *gene-specific and gene-family tags, as well as for primers tagging predicted smallRNA precursors. FLAGdb^++ ^provides access to probe specificities, to primer sequences and to updates of their relationships with gene annotation. The management of Arabidopsis micro-array probes has been extended to other transcriptomic resources. Indeed, FLAGdb^++ ^also integrates the oligonucleotide sets of the Affymetrix ATH1 GeneChip, the probes of two tiling-arrays of different resolutions [26] and the PCR probes of the promoter-dedicated array SAP [27]. The support for these resources allows us to (i) manage the dynamic relationships between micro-array probes and gene annotation, thus facilitating the biological interpretation of differentially expressed gene lists, and (ii) propose interactive links to transcriptomic databases and tools, *i.e. *Genevestigator [28], eFP Browser [29] and CATdb [30].

Gene classification is another major topic in FLAGdb^++^. The different Gene Ontology categories [31] and the detection of conserved protein motifs using the HMM profiles available in PFAM [32] are used to define connections between genes in the four genomes. Furthermore, the integration of expert manual annotation on a selection of gene families provides original information about their organisation, structure and function [33]. For instance, the large pentatricopeptide repeat (PPR) family, involved in the maturation of mitochondrial and plastidial transcripts, has been characterized in detail. This involves 451 Arabidopsis and 477 rice genes, and includes the checking, and correction, of intron-exon structures as well as the organization of the six protein motifs, the complexity of which is a particularity of the family [34,35]. The FLAGdb^++ ^database also contains the location and classification of all the Arabidopsis genes that encode transcription factors, comprising 2,182 genes distributed among 75 distinct families. Similarly, we have integrated 31,876 transposable elements (mainly relics) annotated using a semi-automatic method based on established reference sets [36] and classified within 327 subfamilies.

Beyond the integration of data, FLAGdb^++ ^also provides cross references and web links to external resources and tools (Table 2). With a selection of more than 20 complementary databases, FLAGdb^++ ^constitutes a structuring portal, helping users to build their functional analysis and data mining approaches.

**Table 2:** External links and cross references

Database	Scope and targets	Website URL
ABRC	Arabidopsis biological resource center	http://abrc.osu.edu/
Arabidopsis-TF	Classification of transcription factors (At)	http://urgv.evry.inra.fr/projects/arabidopsis-TF/
Aramemnon	Membrane protein database (At, Os)	http://aramemnon.botanik.uni-koeln.de/
ATOMEdb	ORFeome resource (At)	http://urgv.evry.inra.fr/ATOMEdb
CATdb	CATMA Transcriptome database (At)	http://urgv.evry.inra.fr/CATdb
eFP Browser	Transcriptome database (At, Os, Pt)	http://www.bar.utoronto.ca/
GABI-Kat	GABI Arabidopsis T-DNA mutants	http://www.gabi-kat.de/
GenBank	DNA and protein repository at NCBI	http://www.ncbi.nlm.nih.gov/
GeneFarm	Manually annotation of families (At)	http://urgi.versailles.inra.fr/Genefarm/
Genevestigator	Transcriptome database (At)	http://www.genevestigator.com
Genoscope	Genoscope Genome Browser (Vv)	http://www.genoscope.cns.fr
IJPB	INRA Arabidopsis insertion mutants	http://dbsgap.versailles.inra.fr/portail/
InterPro	Classification of protein families	http://www.ebi.ac.uk/interpro/
JGI	DOE Joint Genome Institut (Pt)	http://www.jgi.doe.gov/
KOG	Clusters of Orthologous Groups (Pt)	http://www.ncbi.nlm.nih.gov/COG/
MAtDB	Arabidopsis genome at MIPS	http://mips.helmholtz-muenchen.de/plant/
PDB	Protein structure Data Bank	http://www.pdb.org/
PFAM	Conserved motifs in protein families	http://pfam.sanger.ac.uk/
RAP-DB	Rice Annotation Project Database	http://rapdb.dna.affrc.go.jp/
SwissProt	Manually annotation of proteins	http://www.uniprot.org/
TAIR	The Arabidopsis Information Resource	http://www.arabidopsis.org/
URGI	INRA Genome Browser (Vv)	http://urgi.versailles.inra.fr/

The cross-references with complementary databases are proposed to the users in the 'Link to...' pop-up menus associated with the concerned features.

## Utility and discussion

**Figure 1:**
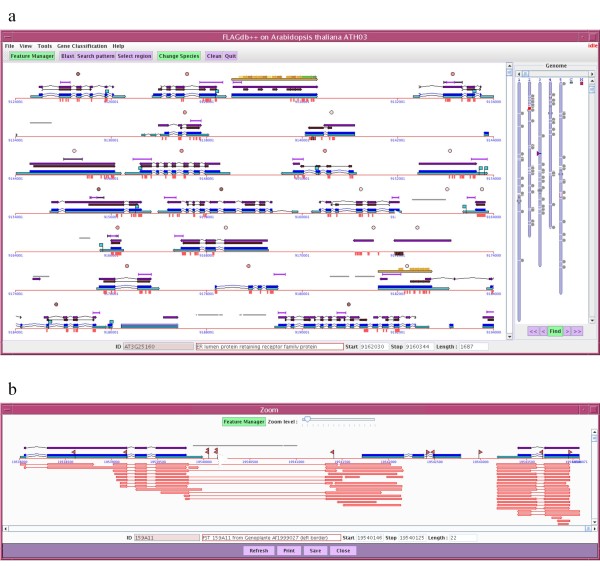
**Screenshots of two windows displaying features in FLAGdb^++^**. (a) In the main window of the FLAGdb^++ ^application, the right panel displays a genome map overview while the left panel shows a detailed local view of 10 Kb in length per line. In this example, the topological organization of the *Harbringer *repeat elements is displayed over the Arabidopsis genome map. The local view presents the following feature tracks: official mRNA and CDS annotation from TAIR (light and dark blue arrows respectively), PFAM motifs (deep brown arrows), alternative CDS annotation from the EuGène predictor (deep purple arrows), CATMA gene specific tags and Affymetrix ATH1 primers for microarray approaches (purple lines and small vertical red boxes respectively), expertized repeat element annotation (light grey arrows), expertized PPR motifs and gene annotation (yellow and orange arrows) and cognate transcript sequences (small pink circles above the genes: darker means a greater number of cDNA/EST). All the features are associated with specific pop-up menus supplying additional information, tools, and/or cross-links with other resources. **(**b) 'Zoom in' upon selection of a specific feature opens a new window displaying additional data (according to the user setting selected through the Feature Manager tool). Represented here are all the splice-aligned cognate transcript sequences, *i.e. *cDNA and Sanger/454 ESTs (pink arrows), and the available mutant line tags, *i.e. *T-DNA flanking sequences (red flags). The display of transcript sequence alignments allows the user to detect eventual erroneous annotation or alternative splicing events, as illustrated in this example.

The main view displayed in FLAGdb^++ ^is of different features spanning the chromosome sequence of the selected species. Each data type is situated on a track with a specific graphical object and colour code. This is a classical representation mode for many genome browsers, however the FLAGdb^++ ^application offers marked differences. For example, an original multi-lined display has been preferred in order to display a large genomic environment in a single view, whilst maintaining an important level of detail (Figure 1) thus allowing access to numerous genes without losing information. This multi-lined solution avoids continual zooming in and out or scrolling actions and therefore makes it easier to study gene organization along chromosomes, such as large gene clusters for instance. Furthermore, FLAGdb^++ ^includes a dual-component interface with an interactive genome-wide view displaying additional information and facilitating access to specific loci (Figure 1) thereby making the detection of localisation bias or syntenic regions straightforward. The chromosomal view allows users to visualize and memorize the topological organisation of repeated sequences, members of gene families, blast results or any other features.

The FLAGdb^++ ^interface system simplifies the navigation from genomic sequences to final protein products through the spliced alignments of transcripts, promoter regions, tagged mutations and protein motifs. Also, predicted models of 3 D protein structures are viewable courtesy of to the embedded KiNG software [37]. The display of additional feature tracks is controlled by the user via the 'Feature manager' tool, avoiding data overload which may cloud their biological interpretation. Clicking on any item reveals pop-up windows showing additional data such as functional annotations, prediction and quality scores, or sources.

**Figure 2:**
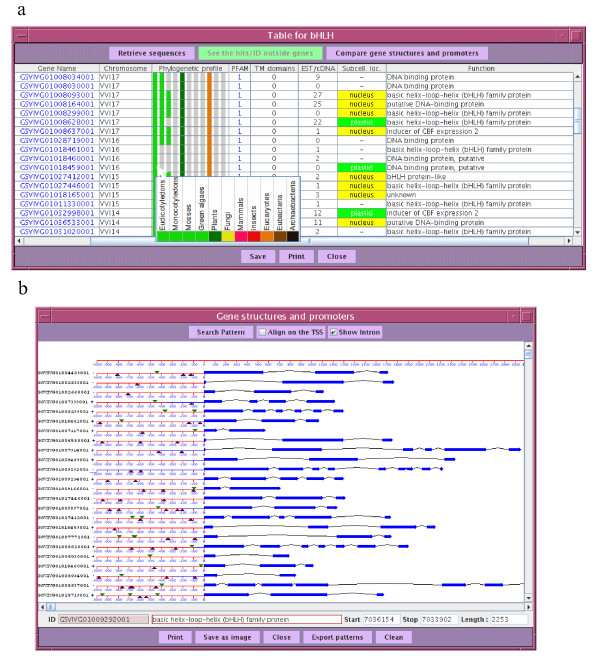
**Display of groups of genes in FLAGdb^++^**. (a) The results of queries using blast, gene lists, keywords, protein motifs, gene families or functional categories, are gathered into tables of functional information (content is defined by the user). These tables interact with the genome browser window and provide cross-links and tools in order to download the data, retrieve sequences (genes, CDS, proteins, promoters relative to ATG or TSS), and to display gene structures (see 2b). Here, the example concerns the bHLH transcription factor family in *Vitis vinifera*. The table presents for each gene, its chromosome, its phylogenetic profile through different phyla (color legend is explained in the pop-up window), the detected PFAM motifs, the number of predicted TM domains, the number of cognate EST/cDNAs, the predicted subcellular localization (scores are available in the pop-up text), and the functional annotation inferred from sequence similarities. (b) A button opens a tool dedicated to gene structures and promoters. The user can remove or sort the genes, choosing whether or not to display the introns, align the structure from ATG or TSS (based on the cognate EST/cDNAs), and look for nucleotide patterns (colored triangles) in the promoter regions.

Aside the ability to access loci through classical queries (such as gene IDs, keywords, sequence similarities, or genomic coordinates), FLAGdb^++ ^also provides tools for exploring the integrated genomes by groups of genes: genes belonging to the same family or to the same GO classification group [31] can be retrieved in a batch with a few clicks of the mouse. Specific interfaces have been developed to allow the selection of a transcription factor or repeat element subfamilies, and also filter GO groups using their evidence code, mirroring the quality and origin of the classification. All these batch queries lead users to synthetic and interactive tables concentrating information on the gene lists: number of cognate transcripts (EST, cDNA, MPSS), presence of T-DNA or transposon mutant lines, phylogenetic profile, functional annotation, subcellular localization, GO terms, PFAM motifs and micro-array probes (Figure 2a). The content of the table of results can be defined by the user and exported in a tabulated text file format. Furthermore, the tables provide a tool for extracting sequences in batches (FASTA format) comprising CDSs, complete genes, proteins or regulator 5' regions defined from the first ATG or the transcription start site. For instance, in order to look for over-represented DNA motifs, which are good candidates for common transcription factor binding sites, such a tool is very useful for retrieving all the promoter sequences from a list of co-expressed genes resulting from a transcriptomic assay. Similarly, for in-depth phylogeny study, all the protein sequences of a gene family are retrievable in a few clicks of the mouse. The tool 'compare gene structures and promoters' graphically displays the structural annotation of a list of genes (Figure 2b), thus facilitating the analysis and characterization of gene families as the user can visually and quickly detect different gene structures within a large group of paralogs, highlighting a possible subfamily, an interesting divergent member or putative erroneous annotations.

**Figure 3:**
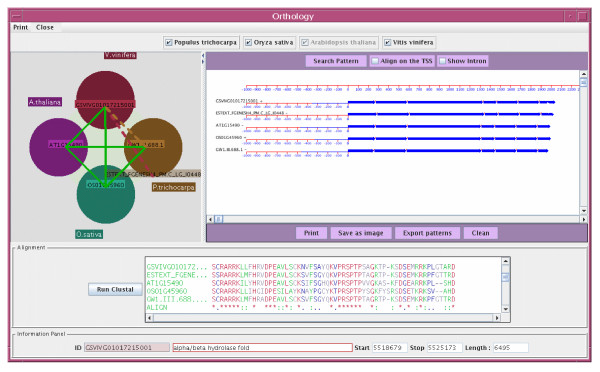
**Screenshot of the 'Orthology' tool**. For any gene in the database, FLAGdb^++ ^displays information about the closest homologous genes in the four integrated species in order to assist the prediction of orthology relationships. The results of all the reciprocal best BLASTP hits (RBH) are displayed together graphically, along with the global protein alignment and intron-exon structures of the genes concerned. In this way, gene structure can also be considered in the prediction of orthologs and eventual erroneous structural annotation (such as gene merging), which render the RBH approach futile, can be easily detected and removed. In this example, all the BLASTP best hits are reciprocal between all genome species (green lines) except between Vitis and Populus genomes.

A recently added tool dedicated to the orthology relationships makes cross-linking between the integrated genomes possible, a particularly powerful feature when inferring function and making comparative analyses. To control whether the BLAST best hits are reciprocal, all against all BLASTP comparisons are graphically represented for a selected gene (Figure 3). Intron-exon structures of candidate orthologous genes are also available for comparison as well as the detection of erroneous annotation. A global protein alignment can be run by launching a Clustal process, whereas the presence of conserved cis-acting regulatory motifs can be tested in the context of a phylogenetic footprinting approach. Numerous other tools are available in the FLAGdb^++ ^application allowing the user to (i) browse the segmental duplications and resulting paralogs of the Arabidopsis genome, (ii) display density curves of features or motifs along the chromosomes, (iii) extract sequences or annotations (GFF, EMBL or GenBank format) between two chromosomal coordinates for external analyses and applications, and (iv) upload private annotations or features and overlay them with the FLAGdb^++ ^data. User preferences are saved at the end of each session, and each graphical object (feature) can be edited in order to prepare relevant figures for use in laboratory books or manuscripts.

We acknowledge the various skill profiles of FLAGdb^++ ^users; they are either biologists or bioinformaticians whishing to address different queries using the database. Some are interested in gene-by-gene or high-throughput approaches, looking for either mutants in their target gene(s) or shared functional characteristics in large co-expressed gene sets. Others are focused on either gene families or large genomic segments for evolution and functional analyses. Since its first release eight years ago, we now have concrete proof of the usefulness of FLAGdb^++^, as it is reflected by its citation in numerous publications (see the website [38]).

## Conclusion

Through a user friendly application, FLAGdb^++ ^offers plant biologists access to a rich array of original genomic resources. JAVA interfaces, combined with intrinsic tools and four annotated complete plant genomes considerably help users to build hypotheses in their translational research or in comparative genomics approaches. Development and integration tasks are directed at highlighting biological correlations between data and speeding up the analyses of groups of genes in a wide range of contexts including genomic regions, gene families or gene function.

We have not described in this paper all the tools and types of display available in FLAGdb^++^. They are however extensively documented on-line [38]. The database is ready for the integration of further plant genomes, dependant of collaborations within the scientific community to provide an equally level of quality as seen in the four presently integrated genomes. The biological data will continue to be updated and enriched through novel experiments, expert works, and results of genomic projects (specifically those concentrated on RNAseq and interactome data), generating further interest in FLAGdb^++ ^within the plant science community over the coming years.

## Availability and requirements

The FLAGdb^++ ^home page [38] provides both access to the installation guide and complete documentation regarding tools and data. To run the FLAGdb^++ ^application, JAVA (JRE version 1.6 or higher) should already be installed on the computer. Database architecture, integrated data and all the pipelines developed (in Perl) to fill the database are available on request for users who want to use the FLAGdb^++ ^environment with other eukaryotic genomes. A Perl script allowing to open the FLAGdb^++ ^application on a specific feature is also available on request in order to create interactive links from other tools or databases. There is no restriction to the use of FLAGdb^++ ^by non-academics.

## List of abbreviations

CDS: coding sequence; GFF: gene feature format; GFT: gene family tag; GO: gene ontology; GST: gene specific tag; FST: T-DNA flanking sequence tag; MPSS: massively parallel signature sequencing; PPR: pentatricopeptide repeat; TPS: terpene synthase; TSS: transcription start site.

## Competing interests

The authors declare that they have no competing interests.

## Authors' contributions

SD, FS, JPT, CG, SG, JCL and PL were involved in the data production, acquisition and/or integration. FS and SD carried out the JAVA software development. FS, VB, JPT and PG were involved in the database conception and management. JPT and AL helped to draft the manuscript. SA coordinated the project and drafted the manuscript. All authors read and approved the final manuscript.
